# Preparation and Characterization of 3D Printed PLA-Based Conductive Composites Using Carbonaceous Fillers by Masterbatch Melting Method

**DOI:** 10.3390/polym11101589

**Published:** 2019-09-29

**Authors:** Rui Guo, Zechun Ren, Xin Jia, Hongjie Bi, Haiying Yang, Tong Ji, Min Xu, Liping Cai

**Affiliations:** 1 Key Laboratory of Bio-based Material Science and Technology (Ministry of Education), Material Science and Engineering College, Northeast Forestry University, Harbin 150040, China; guorui0527@163.com (R.G.); yourong_rzc@163.com (Z.R.); 18437959095@163.com (X.J.); bihongjie1016@163.com (H.B.); yhy1258000@163.com (H.Y.); jtjy5116@163.com (T.J.); 2 Mechanical and Energy Engineering Department, University of North Texas, Denton, TX 76201, USA; Liping.Cai@unt.edu; 3 College of Materials Science and Engineering, Nanjing Forestry University, Nanjing 210037, China

**Keywords:** polylactic acid, polymeric composites, masterbatch melting method, graphene nanoplatelets, electrical conductivity, fused deposition modeling

## Abstract

This study was aimed at improving the conductivity of polylactic acid (PLA)-based composites by incorporating carbonaceous fillers. The composites with the addition of graphene nanoplatelets (rGO) or multi-walled carbon nanotubes (MWCNTs) were fabricated by the masterbatch melting method in order to improve the dispersion of the two kinds of nano-fillers. The results showed that, with the addition of 9 wt % rGO, the volume electrical resistivity of the composite reached the minimum electrical resistance of 10^3^ Ω·m, at which point the conductive network in the composites was completely formed. The interfacial compatibility, apparent viscosity, and the thermal stability of the composite were also good. The rGO functionalized by sodium dodecylbenzene sulfonate (SDBS) was an efficient method to further improve the electrical conductivity of the composite, compared with tannic acid and MWCNTs. The resistivity was reduced by an order in magnitude. Patterns printed onto different baseplates by fused deposition modeling illustrated that the functionalized composite had certain flexibility and it is suitable for printing complex shapes.

## 1. Introduction

Conductive polymer composites with better performances and multifunction, typically composed of polymer matrix and carbonaceous fillers [1], are promising for applications in many potential fields [2], such as electronics devices, thermal management parts, etc. [3,4]. The carbonaceous fillers became an attractive research area in investigating composite applications since their invention [5]. In the past several years, with the growing requirement of conductive polymer composites, the research of carbonaceous fillers was of great importance [3]. Carbonaceous fillers, including graphene [6], carbon nanotubes [7,8], graphite [9], and carbon fiber [10,11], are supposed to be one of the most prospective fillers for incorporation into the polymer matrix because of their intrinsic characteristics of light weight, and high electrical and thermal conductivity [12]. Among the carbonaceous fillers, graphene, which is a two-dimensional atomic layer of *sp*^2^-hybridized carbon atoms arranged in a honeycomb lattice [13,14,15,16,17], is drawing more and more attention because of its superb mechanical, electrical, and thermal properties [18].

Recently, some efforts were made to apply conductive polymer composites to the fused deposition modeling (FDM) technology [19,20,21], because additive manufacturing was proven to overcome the limitations of conventional procedures in the production of complex geometries [18]. With the increasing attention of environmental issues, instead of petroleum-based polymers, polylactic acid (PLA) was chosen as the matrix of the conductive polymer composite suitable for FDM due to its biodegradability and environmental friendliness [22].

This study aimed to improve the electrical conductivity of PLA-based composites by incorporating carbonaceous fillers, especially by adding graphene nanoplatelets. Graphene nanoplatelets are easy to agglomerate owing to their inherent π–π stacking interaction [3], which may cause their poor dispersion in a PLA matrix [23]. To solve this problem, the method of masterbatch-based melt blending was used for the preparation of composites. To improve the brittleness of PLA, thermoplastic polyurethane (TPU) was used in the composites [24]. In order to further enhance the electrical conductivity of the composites, the redox graphene nanoplatelets (rGO) were also non-covalently functionalized by tannic acid (TA), sodium dodecyl benzene sulfonate (SDBS), and multi-walled carbon nanotubes (MWCNTs).

## 2. Materials and Methods

### 2.1. Materials

The rGO platelets were obtained from Tianyuanda Graphite Co., Ltd. (Qingdao, China), while multi-walled carbon nanotubes (XFM22) were supplied by Nanjing XFNano Material Technology Co., Ltd. (Nanjing, China). The impurity of multi-walled carbon nanotubes was less than 5%, in which the number of walls was 23–25. PLA (4032D) and thermoplastic polyurethane (TPU, 85A) were, respectively, purchased from NatureWorks, Blair, NE, USA and BASF, Ludwigshafen, Germany. Three kinds of solvents, *N*,*N*-dimethyl formamide (DMF), dichloromethane (DCM), and ethanol were all obtained from Tianjin Fuyu Chemical Co., Ltd., Tianjin, China. Tannic acid (TA) and sodium dodecyl benzene sulfonate (SDBS) were separately purchased from Tianjin Fushen Chemical Reagent Co., Ltd. (Tinajin, China) and Tianjin Tianli Chemical Reagent Co., Ltd. (Tianjin, China).

### 2.2. Sample Preparation

**Figure 1:**
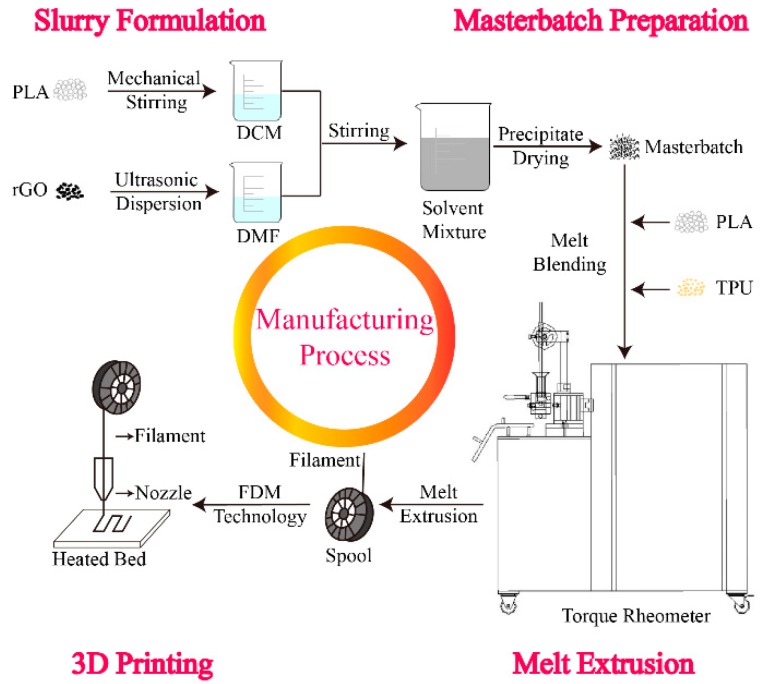
Main steps of the composite preparation process.

PLA and TPU, with a mass ratio of 7:2, were dried, respectively, at 50 °C for 8 h and 103 °C for 12 h before being used. In order to make the carbonaceous fillers disperse better, the composites were prepared in a two-step process. Main steps of the composite preparation process are shown in Figure 1. Firstly, the masterbatch was prepared during solution mixing. Among different composite fabrication methods, the method of solution mixing was considered to be an easier way to ensure good homogeneity at a laboratory scale [25]. Then, 20 wt % PLA was added into DCM and stirred for 12 h mechanically. Carbonaceous fillers (rGO or MWCNT), with a mass ratio of 6, 9, and 12 wt %, were separately added to DMF at a concentration of 2 mg/mL and ultrasonically dispersed for 1 h using a sonicator (Ningbo Xinzhi Biotechnology Co., Ltd., Ningbo, China) with a pause model of 1000 W. Then, the two kinds of solution were mixed under mechanical stirring. The solvent mixture was precipitated into ethanol, filtered, and dried at 40 °C for 24 h in a drying oven. Another step was melt-blending. The masterbatch was melt-mixed with the remaining PLA and TPU by a torque rheometer (HAAKE PolyLab OS, Vreden, Germany). Considering the high cost of the carbonaceous nano-fillers, granules obtained were used for characterization. The control composite was made in the same way, but no carbonaceous fillers were added. Composites with different rGO contents (0, 6, 9, and 12 wt %) were marked as rGO-0, rGO-6, rGO-9, and rGO-12, respectively.

The optimum type and the content of carbonaceous fillers were determined by the above experiment, and then rGO was non-covalently functionalized by TA, SDBS, and MWCNTs. The modifiers and rGO were added into the solution of DMF. The mass ratios of rGO/TA, rGO/SDBS, and rGO/MWCNT were 2:1, 5:1, and 3:1, respectively, which were added into the composites. Composites with different modifiers were marked as rGO, rGO-TA, rGO-SDBS, and rGO-MWCNT. After optimizing the formulation, the granules obtained by the torque rheometer were fed into a single-screw extruder to produce filaments with a diameter of 1.75 mm ± 0.01 mm, which were applied to FDM. The extruder (SHSJ25), with a screw length-to-diameter ratio (L/D) of 12:1, a screw speed of 35 Hz, and a draw speed of 17 Hz, was manufactured in Dongguan Songhu Plastic Machine Co., Ltd. in China. The temperatures for each stage were pre-set at 160, 190, and 160 °C (from the feeder to the mold). The temperature of the cooling water was 55 °C. At last, the parts were printed by an FDM printer (HORI Z400D, Beijing Hui Tianwei Technology Co., Ltd., Beijing, China). The parameters used for the FDM printer are listed in Table 1.

### 2.3. Characterization

#### 2.3.1. Electrical Resistivity

The volume electrical resistivity of the composites was tested by the four-probe method (ST2722, Suzhou Jingge Electronics Co., Ltd., Suzhou, China). When the resistivity was higher than 2.0 × 10^7^, the resistivity of the composite powder was tested by a ST2643 high-resistance meter.

#### 2.3.2. Scanning Electron Microscopy (SEM)

The morphology of the composites was observed by the JSM-7500F SEM (FEI QUANTA 200, Eindhoven, The Netherlands). All samples were fractured in liquid nitrogen, and then the fracture surfaces were gold-sprayed before the observation.

#### 2.3.3. Rheology Properties

The rheological measurements were conducted using a HAAKE Torque Rheometer (PolyLab OS, Vreden, Germany) with a roller rotor. The temperature was set at 190 °C, and the speed of the rotor was 50 r·min^−1^.

#### 2.3.4. Differential Scanning Calorimetry (DSC)

The DSC analysis was characterized by a DSC Q20 (TA Instruments, New Castle, DE, USA). The test was divided into three steps. In the first step, the samples were heated from −20 to 200 °C and stabilized at 200 °C for 5 min to eliminate the effect of thermal history. In the second step, the samples were cooled from 200 to −20 °C and held at −20 °C for 1 min. Finally, the samples were heated from −20 to 200 °C and kept at 200 °C for 1 min. Both the heating rate and the cooling rate were set at 10 °C·min^−1^. All scans were carried out under the nitrogen atmosphere. The crystallinity (*X_c_*) of the composites could be calculated by Equation (1).

(1) $$X_{c}\left(\%\right)=\frac{\Delta H_{m}}{\varphi_{PLA}\times \Delta H_{m}^{0}}$$

where Δ*H_m_* is the enthalpy of fusion (J/g), *φ_PLA_* is the actual weight fraction of PLA in the composites, and ${\Delta H}_{m}^{0}$ is the standard enthalpy of PLA for 100% crystallinity, equal to 93.7 J/g.

#### 2.3.5. Thermalgravimetric Analysis (TGA)

The thermal stability was examined by the Thermalgravimetric Analyzer (Q50, TA Instruments, New Castle, DE, USA) from 30 to 600 °C, with a heating rate of 10 °C·min^−1^. All tests were carried out under nitrogen atmosphere.

#### 2.3.6. X-ray Diffraction (XRD)

The XRD analysis was accomplished with an X’ Pert3 Powder X-ray diffractometer (PANalytical, Almelo, The Netherlands). Cu Kα radiation (λ = 1.541 Å) was used. The scattering angle range of 2θ was from 5° to 45° with a scan rate of 5°·min^−1^. 

#### 2.3.7. Fourier-Transform Infrared Spectroscopy (FTIR)

The FTIR spectra, measured with the FTIR instrument (Nicolet 6700, Waltham, MA, USA), were collected from 600 to 4000 cm^−1^. The obtained particles were directly used for the testing, and the test mode was selected as the attenuated total reflectance (ATR) mode.

## 3. Results and Discussion

### 3.1. Effect of the Carbonaceous Filler Types on PLA-Based Composites

#### 3.1.1. Electrical Resistivity of the PLA-Based Composites

**Figure 2:**
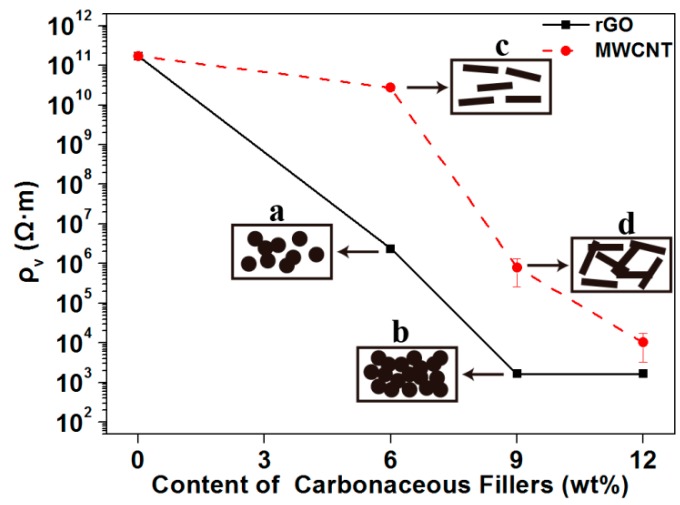
Volume electrical resistivity of the composites at different loadings of redox graphene nanoplatelets (rGO) or multi-walled carbon nanotubes (MWCNTs).

Figure 2 shows the volume electrical resistivity of the composites as a function of the carbonaceous filler content. The black line represents the volume electrical resistivity of the composites with the addition of rGO, while the red dashed line represents the addition of MWCNT. It can be clearly seen in Figure 2 that the volume electrical resistivity of the composite significantly decreased upon adding rGO. When 6 wt % rGO was added, the volume electrical resistivity reduced by five orders of magnitude, going from 10^11^ Ω·m (rGO-0) to 10^6^ Ω·m (rGO-6). With the addition of 9 wt %, the magnitude of volume electrical resistivity reached the lowest value, which was 10^3^ Ω·m. When rGO was continuously added to the composites until reaching the 12 wt % content, the resistivity of the composites no longer decreased in magnitude. Then, as shown by the red line, with the addition of MWCNTs, the volume electrical resistivity of the composites decreased. When the content of MWCNTs increased to 9 wt %, the volume electrical resistivity of the composite dropped significantly, reaching the order of 10^5^ Ω·m. As MWCNTs were continually added into the composites, the volume electrical resistivity decreased slightly, reduced by an order of magnitude (from 10^5^ to 10^4^ Ω·m).

**Figure 3:**
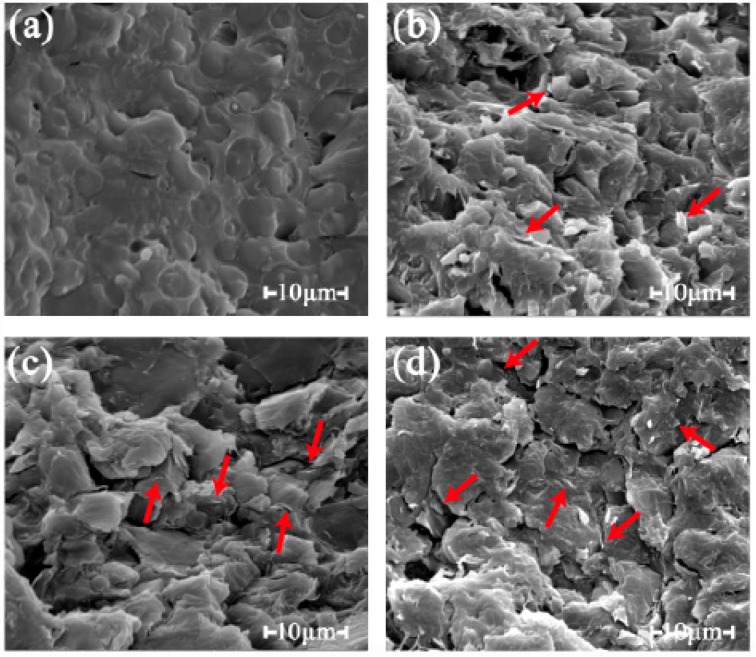
SEM micrographs of the fracture surfaces of composites with different rGO content: (**a**) 0 wt % rGO added in the composite, (**b**) 6 wt %, (**c**) 9 wt %, and (**d**) 12 wt %.

**Figure 4:**
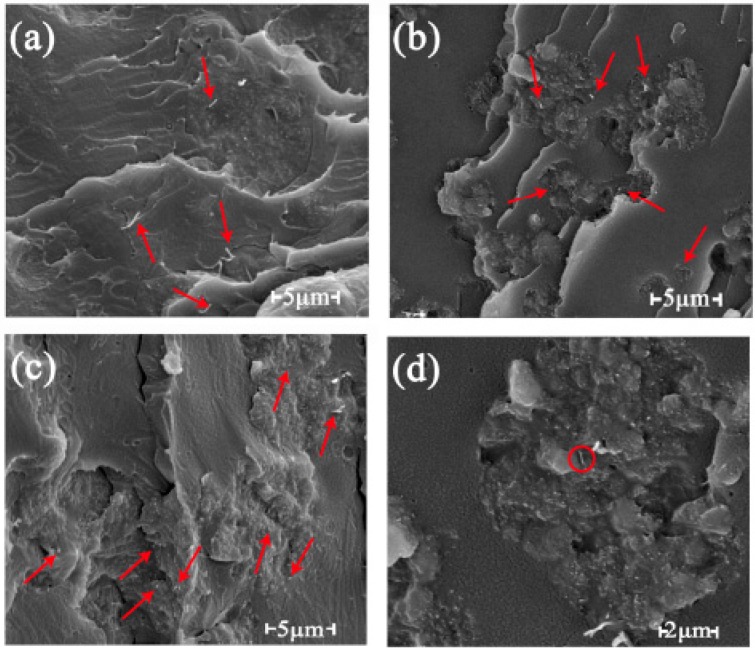
SEM observation of the fracture surfaces of composites with different MWCNT content: (**a**) 6 wt %, (**b**) 9 wt %, and (**c**) 12 wt %; (**d**) the 10,000× magnification SEM image of the composite with 9 wt % MWCNTs.

The conductive mechanism of the composites is shown in the rectangular boxes in Figure 2. The main reason that affected the electrical conductivity of the composites was the random orientation of the fillers during processing, which would determine the formation of the conductive network in the composites [2]. When a small amount of carbonaceous fillers was added, the volume electrical resistivity of the composites remained at the same order of magnitude. At this point, the fillers were scattered in the matrix, but not in contact with each other (shown as c in Figure 2). As the filler content increased, the volume electrical resistivity of the composites decreased exponentially with an order of magnitude. This was because the increase in the content of the fillers in the matrix caused the fillers to interact with each other, and the conductive path in the composites began to form (shown as a or d in Figure 2). When the filler content was continually increased, the volume electrical resistivity of the composites continually decreased, reaching the minimum. Moreover, as the content increased, the volume electrical resistivity of the composites no longer changed in magnitude. When the content of the fillers in the matrix reached a certain concentration, the conductive network in the composites was completely formed, resulting in the lowest volume electrical resistivity (shown as b in Figure 2). Additionally, this phenomenon can be verified by the microscopic morphology of the composites in Figure 3 and Figure 4 as below.

#### 3.1.2. Morphology of the PLA-Based Composites

The red arrows in Figure 3 indicate that the rGO was dispersed in the matrix. The rGO was presented as sheet-like flakes with sharp edges. With the growing content, an increased amount of rGO was observed in the field of view at the same magnification. Moreover, the rGO nanoplatelets were uniformly dispersed and tightly embedded in the matrix without agglomeration, which proved the good compatibility and the mechanical interlocking caused by the strong interfacial adhesion between the rGO and the matrix [26,27]. However, the composites with the addition of the rGO (Figure 3b–d) gained a rougher fracture surface due to the special structure of the rGO nanoplatelets with sharp edges, compared with the smooth fracture surface of the composite without rGO (Figure 3a) [28].

In Figure 4, the red arrows point to the small tubes or white dots, showing MWCNTs in the composite. Carbon nanotubes are one-dimensional nano-fillers that are easy to agglomerate and entangle with each other due to their small diameter and high surface energy [29]. Because this affected their dispersion in the matrix, the MWCNTs were ultrasonically dispersed prior to the preparation of the composites. Figure 4 shows that the MWCNTs were uniformly dispersed in the matrix, and the ends of some MWCNTs were buried into the matrix and were partially pulled out. The interfacial adhesion between the MWCNTs and the matrix was good. Although some sections of the MWCNTs were pulled out of the matrix, there was no obvious separation between the MWCNTs and the matrix because of the van der Waals force between the MWCNTs and the matrix, allowing them to form a stable compatible structure [30]. The conductivity mechanism of the MWCNT/PLA composites was the same as that of the rGO/PLA composite. When the MWCNT content was less than 6 wt %, the MWCNTs were uniformly distributed in the matrix but separated from each other (shown in Figure 4a); at this point, the conductive pathway in the composite was blocked by the polymer matrix. As the MWCNT content in the composites increased to 9 wt %, the MWCNTs began to interact with each other, and, at that point, the conductive path started to form. When the loading of MWCNT increased to 12 wt %, a more complete conductive network and a denser structure were already formed between MWCNTs [12]. The 10,000× magnification SEM image of the composite with 9 wt % MWCNT is shown in Figure 4d. It can be seen from Figure 4d that the MWCNT in the red circle may be in contact with other MWCNTs, which could be proof that conductive fillers can contact each other at a relatively high content.

### 3.2. Effect of rGO Content on the PLA-Based Composites

By comparing the effect of the two carbonaceous fillers on the electrical conductivity of PLA-based composites, rGO was selected as the conductive filler of the composites, in order to achieve better conductivity composites with a small addition amount of rGO.

#### 3.2.1. Rheological Properties

**Figure 5:**
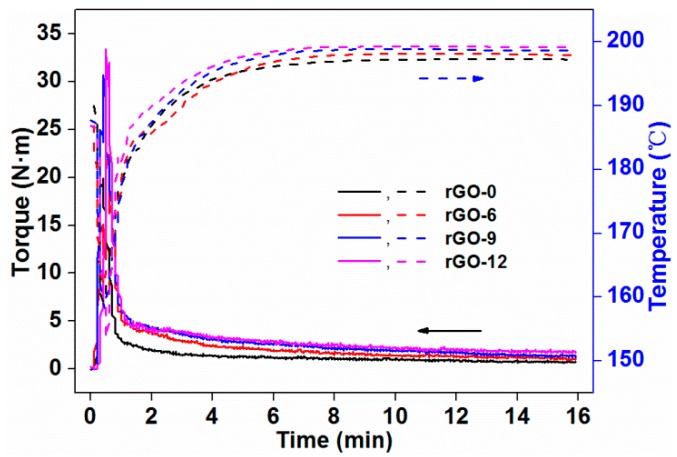
Plots of torque and temperature as a function of time filled with different content of rGO.

Figure 5 reflects the effect of the rGO content on the torque rheological properties of PLA-based composites. The solid lines in the figure represent the curves of torque versus time, and the dashed lines represent the curves of temperature versus time. The torque of the composites increased rapidly when the pellets were added to the mixing cavity, as the cold solid particles impeded the free rotation of the rotor [31]. Then, the torque quickly reached the maximum, and a feeding peak appeared on the plot. Under the synergistic effect of the heating and the shearing of the rotors, the pellets were heated and began to melt, which led to a gradual reduction of the torque. Then, it reached the equilibrium torque, and, at that point, the pellets were uniformly mixed. The equilibrium torque reflected the indirect apparent viscosity and the flow characteristics of the melt system [32]. As the equilibrium torque decreased, the viscosity of the system decreased as well. At this time, the system gained a better fluidity. The change in temperature can also be seen in Figure 5. With the addition of pellets, the temperature in the mixing cavity decreased firstly, as the pellets were cold. Then, the temperature rose when the pellets began to absorb heat and melt. The final temperature in the cavity was in excess of the set value, which was equal to 190 °C, due to the heat generated by shear friction. When the particles were mixed evenly, the temperature tended to be stable and no longer rose.

The equilibrium torque (*T*_e_) was calculated from the average of the last three minutes of torque. The shear heat (Δ*T*) was equal to the difference between the average temperature of the last three minutes and the set temperature. The results are shown in Table 2. With the addition of rGO, the equilibrium torque of the system increased, which meant that the fluidity of the system deteriorated and the apparent viscosity increased. The increase in the viscosity of the system made the rotors difficult to rotate, resulting in an increase in the shear heat.

#### 3.2.2. Thermal Stability

**Figure 6:**
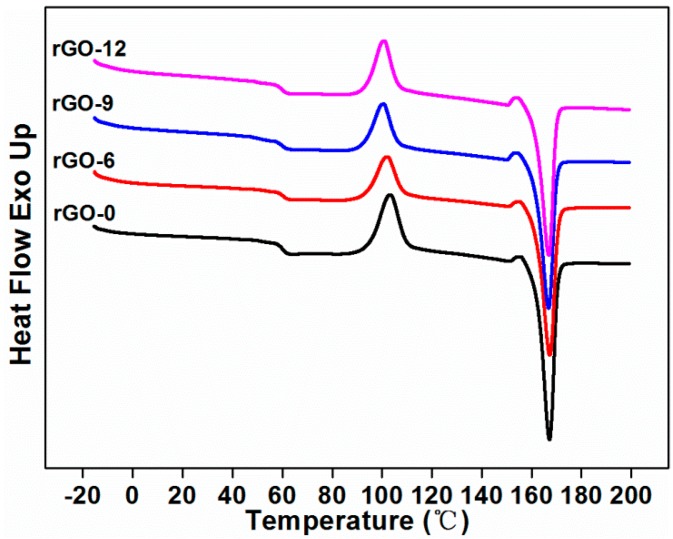
Second melting differential scanning calorimetry (DSC) curves of composites with varying rGO content.

**Figure 7:**
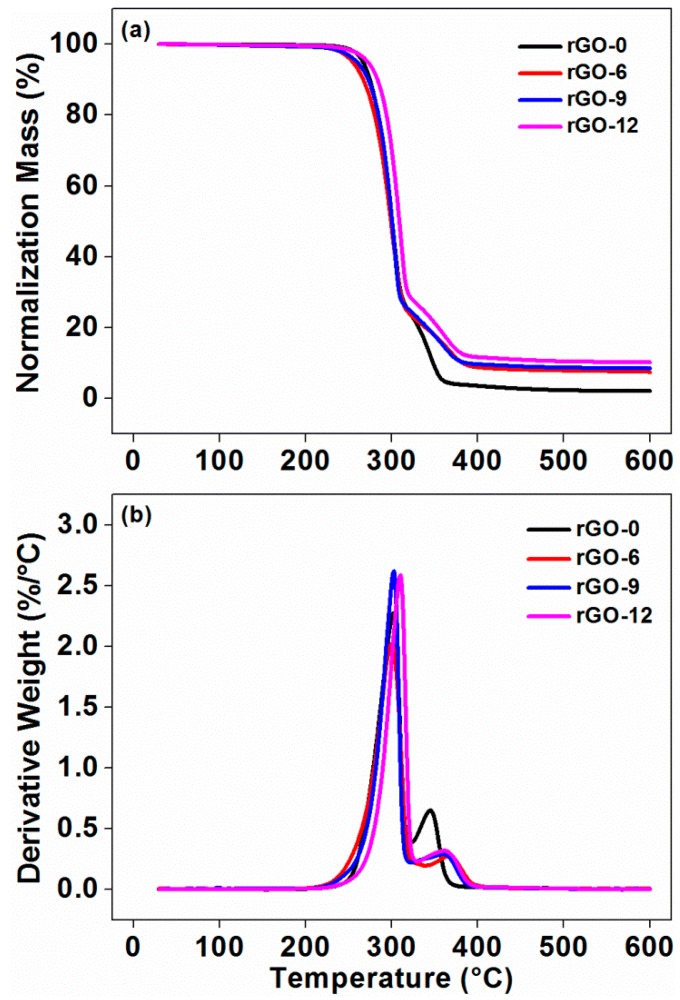
Thermogravimetry (TG) (**a**) and differential TG (DTG) (**b**) curves of the composites with different rGO contents.

To determine the effect of rGO on the thermal properties of the composites, DSC and TGA tests were completed, and the results are shown in Figure 6 and Figure 7, respectively. Characteristic values of the two tests are displayed in Table 3. The glass transition of the composite without rGO started at 60.35 °C, the cold crystallization occurred at 103.35 °C, and a melting peak appeared at 167.11 °C. Since TPU is an amorphous polymer, the crystallization behavior of the composites was attributed to the PLA component in the matrix. On the left side of the sharp and narrow high temperature melting peak, a weak exothermal peak can be observed, which may be attributed to the transition of α’ crystals produced during the cold crystallization to α crystals [33]. The crystal transition at this time can also be considered as recrystallization, in which small imperfect crystals were converted into more stable crystals [26]. The specific values of the composites with different rGO contents are shown in Table 3.

As shown in Table 3, with the increasing content of rGO, there was a small drop in the glass transition temperature, indicating that miscibility occurred in the matrix, despite the incompatible structure of rGO and PLA [34]. The melting temperature of the composites remained comparable, which implied that the printing parameters for FDM technology were applicable to all composites with or without rGO. The cold crystallization temperature of the composites shifted to a lower temperature after adding rGO due to the nucleation effect of rGO [35], which can provide heterogeneous nucleation sites for PLA, thereby improving the cold crystallization ability of PLA [26]. With the addition of rGO, the crystallinity of the composites decreased. This was because, when the thermal conductive fillers were added, the thermal conductivity of the composites increased to accelerate the heat conduction, which was adverse to the crystallization [36]. In addition, the well-dispersed nano-fillers could form a physical barrier to the PLA crystals, thereby inhibiting the crystallization and resulting in a decrease in the crystallinity of the composites with the loading of rGO.

The curves and values of TGA are shown in Figure 7 and Table 3, respectively. As shown in Figure 7, the decomposition of the composites was divided into two stages. The first stage corresponded to the decomposition of the component PLA at about 301.97 °C, while the second stage stood for the decomposition of TPU in the matrix, which was at about 344.63 °C. Comparing rGO-6 with rGO-0, the decomposition temperature of PLA was almost unchanged, but the decomposition temperature of TPU increased from 344.63 to 365.95 °C. When rGO was continually added into the composites (rGO-9 and rGO-12), the decomposition temperature of PLA increased, the decomposition temperature of TPU decreased, and the difference between the two values gradually became smaller, which can be explained by the interfacial interactions between rGO and the matrix [28]. When the composites began to be heated, free radicals were generated, leading to the degradation of the matrix; nevertheless, rGO can be combined with free radicals by conjugation to retard the degradation process [37,38]. When taking the epitaxial onset temperature (*T*_onset_) into account, the temperature of rGO-6 was lower than rGO-0, which may be attributed to the acceleration of thermal diffusion in the matrix by adding a phase of the thermally conductive rGO [39]. As the rGO content increased, *T*_onset_ of the composites shifted to a higher temperature. Compared with rGO-0, *T*_onset_ of rGO-12 was 289.52 °C, increased by 11.03 °C, suggesting that the degradation of the matrix was retarded effectively. The enhancement of the thermal stability of the composites by incorporating rGO was mainly because of the uniformly dispersed rGO with a special layered structure, which can be an effective thermal barrier for the matrix [40]. Meanwhile, the strong interfacial interaction between rGO and the matrix can also restrict the mobility of the PLA chains near the surface of rGO; thus, the composite can gain excellent thermal stability [41]. After being heated to 600 °C, the residue of the composites increased with the incorporation of rGO, which further demonstrates that the introduction of rGO improved the thermal stability of the composites. The residue of the composites at 210 °C (the highest temperature during processing and additive manufacturing) was higher than 99.20%, indicating that the composites hardly decomposed at or below 210 °C. Thus, the composites were not seriously decomposed and damaged during the processing, which further proved that 210 °C was a suitable temperature for FDM technology.

#### 3.2.3. XRD Results

**Figure 8:**
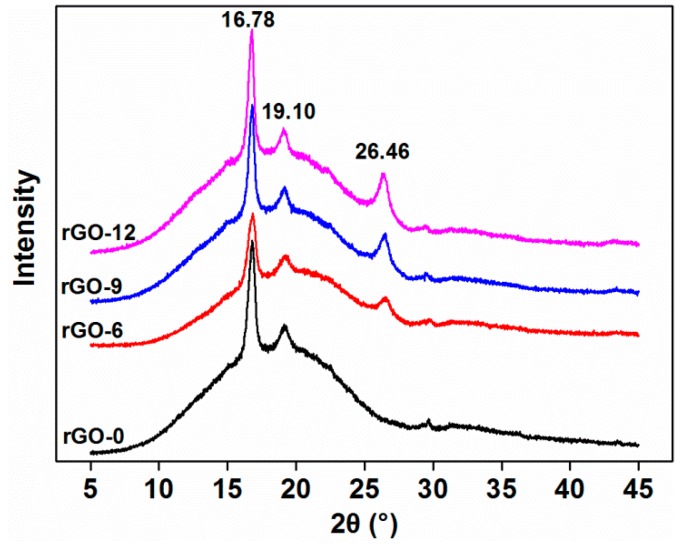
X-ray diffraction (XRD) patterns of the composites with varying rGO content.

The XRD results are shown in Figure 8 for the composites from 5° to 45°, which can reflect the crystal structure behavior of the composites. All composites exhibited two typical characteristic peaks at 2θ = 16.78° and 19.10°, which corresponded to the diffraction of the α crystal form of PLA at the (100/200) and (203) crystal planes [12,42]. The adding of rGO somewhat affected the crystalline form of PLA in the composite. The crystal plane of PLA did not change; however, the intensity of the diffraction peaks at 16.78° decreased firstly and then increased with the addition of rGO. This was consistent with the DSC results. By incorporating rGO, a characteristic diffraction peak appeared at 2θ = 26.46°, which was attributed to the crystalline form of the stacked rGO nanoplatelets [43,44]. This demonstrated that rGO can maintain a good crystalline form in the composite [12]. As the concentration of rGO went up, the intensity of the diffraction at peak 26.46° enhanced, indicating that rGO began to exist as a continuous phase and tended to form dense clusters, which was beneficial for the electrical conductivity of the composite [34].

### 3.3. Properties of the Modified Composites

**Figure A1:**
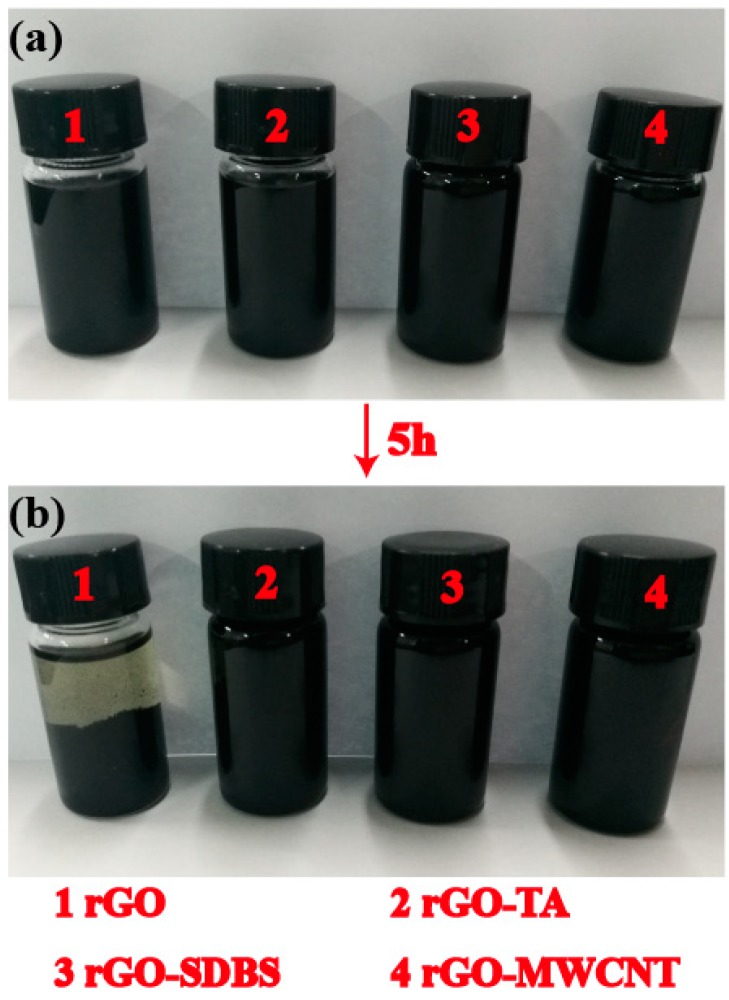
Photos of slurry after sonication at (**a**) 0 h and (**b**) 5 h (1—rGO, 2—GO–tannic acid (TA), 3—GO-SDBS, and 4—GO-MWCNT).

Taking the good electrical conductivity and thermal stability into consideration, rGO was selected as the conductive filler for the composites, and the addition amount was chosen to be 9 wt %, which was used for subsequent research. In order to further improve the conductivity of the composites, TA, SDBS, and MWCNTs were used for non-covalent functionalization of rGO. The stability of the slurry after the functionalization of rGO was shown in Figure A1.

#### 3.3.1. FTIR Results of the Masterbatch

**Figure 9:**
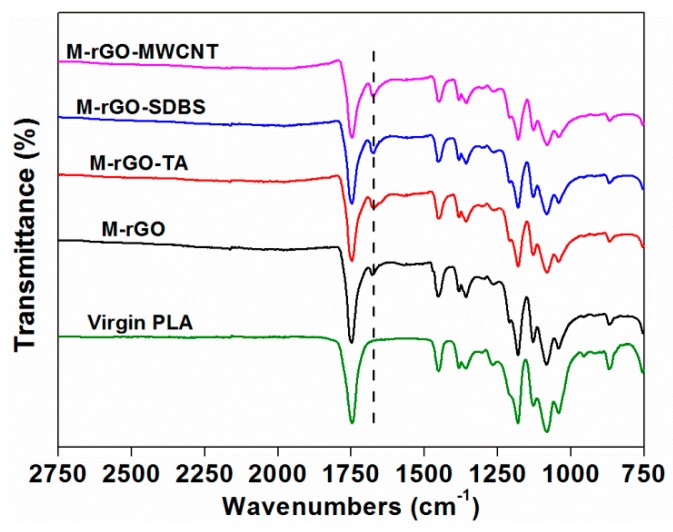
Fourier-transform infrared (FTIR) spectrum of the virgin polylactic acid (PLA) and the composites with the modifiers.

Figure 9 shows the results of the FTIR analysis. A new characteristic peak of the masterbatch with rGO appeared at 1676 cm^−1^, which represented the stretching vibration of C–C. The peak of the masterbatch by incorporating the modifiers became broad, implying that hydrogen-bond interactions were generated between the matrix and the modified fillers [28]. The characteristic peaks of the PLA presented in the masterbatch covered those of the modifiers; thus, it could be indirectly proven by this method that the modifiers were successfully adsorbed onto the rGO. Moreover, the peak intensity at 1676 cm^−1^ enhanced, indicating that the addition of the modifiers facilitated the interfacial interactions of rGO and the matrix.

#### 3.3.2. Electrical Resistivity of the rGO/PLA Composites

**Figure 10:**
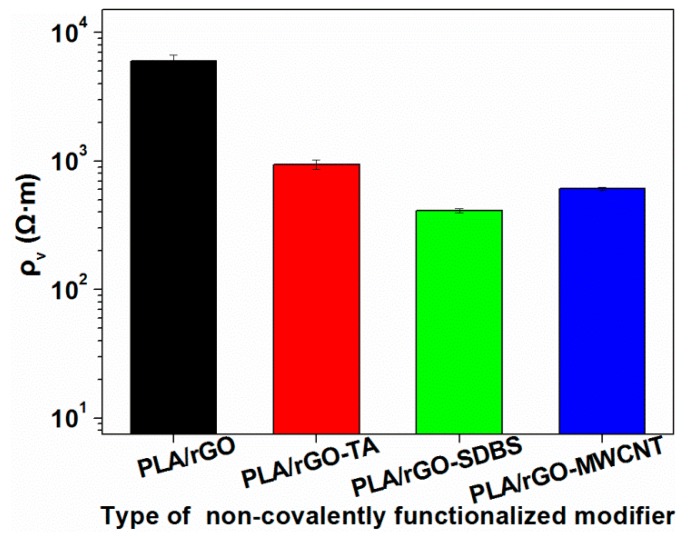
Electrical resistivity of the modified composites.

Figure 10 shows the different electrical resistivities of the modified composites. The addition of rGO-TA or rGO-SDBS slightly reduced the electrical resistivity of the composite. The incorporation of rGO-SDBS can be a more effective way to reduce the electrical resistivity of the composites, dropping the electrical resistivity by an order of magnitude. This may be attributed to the improved interfacial compatibility by the good dispersion of rGO-SDBS.

#### 3.3.3. SEM Photographs and Energy-Dispersive Spectroscopy (EDX) Spectra of the rGO-SDBS/PLA Composites

**Figure 11:**
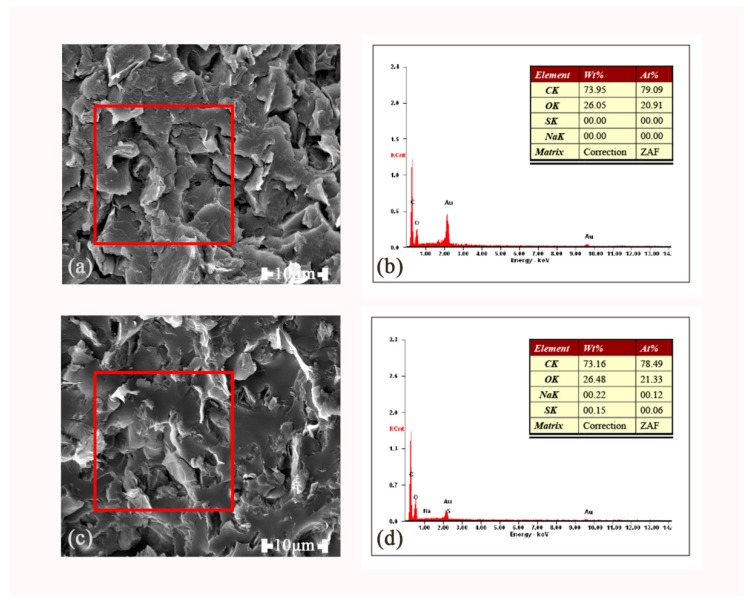
SEM images of rGO (**a**) and rGO–sodium dodecylbenzene sulfonate (SDBS) (**c**) and energy-dispersive spectroscopy (EDX) spectrum of rGO (**b**) and rGO-SDBS (**d**).

Figure 11 shows the morphology and EDX spectrum of rGO and rGO-SDBS obtained by the SEM examination. It can be obviously seen from the figure that the voids and gaps in rGO-SDBS were less than in rGO, which proved that the compatibility between the rGOs modified by SDBS and the matrix were improved. The red square in the images denotes the region of the X-ray scan spectrum. The data in the table indicate the relative mass fraction and atomic percentage of specific elements in the composites before and after the modification, respectively. Unlike the matrix and rGO, two kinds of elements S and Na were contained in SDBS. Both elements could be detected in the composites with rGO-SDBS. The relative mass fraction of S was 0.15% and that of Na was 0.22%, which further proved that the rGO was successfully non-covalently functionalized by SDBS.

#### 3.3.4. Thermal Stability of the rGO-SDBS/PLA Composites

**Figure 12:**
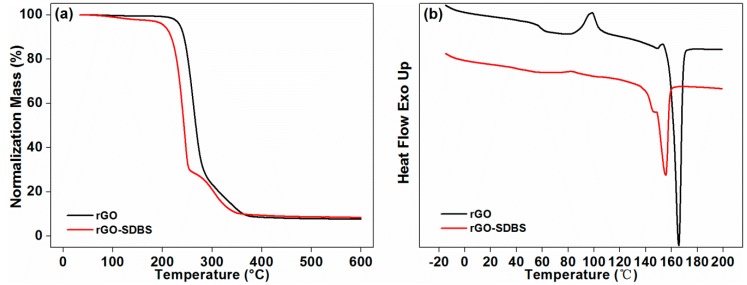
TGA curves (**a**) and DSC curves (**b**) of the composites before and after functionalization.

TGA and DSC results are plotted in Figure 12a,b, respectively. As shown in Figure 12a, there was a thermal weight loss in rGO-SDBS around 100 °C, which represented the evaporation of moisture in the composite. Although the composite was stored in a sealed container before the testing, the samples may absorb humidity from the atmosphere during the process of preparation in the test [45]. This was mainly because moisture can be easily absorbed by SDBS, and the composite after the introduction of SDBS exhibited a similar behavior. It was also evidence for the successful functionalization. However, the effect of moisture (less than 1.3% mass loss) was not statistically significant. Therefore, it insignificantly affected the performance of the composites. The residue of rGO-SDBS at 600 °C increased by 10% compared with that of rGO, which indicated that the thermal stability of the composites was improved. It can be clearly seen in Figure 12b that a double melting peak at a lower temperature can be observed in the composite after adding SDBS. The appearance of the double melting peak was mainly caused by the melting of the crystals formed in the cold crystallization stage during the heating and the recrystallization during the melting at a higher temperature [46]. In the recrystallization stage, different crystalline forms may be generated [26,46].

### 3.4. Models of PLA/rGO-SDBS Composites by FDM

**Figure 13:**
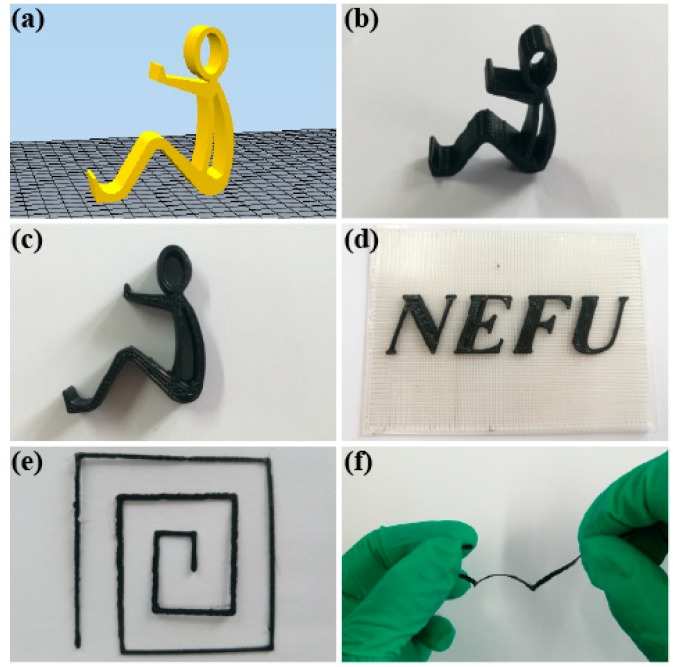
Different models printed by fused deposition modeling (FDM): the human-shaped phone stand holder from blueprint to the finished product (**a**–**c**), NEFU (the abbreviation of Northeast Forestry University) printed on the PLA baseplate (**d**), the printed circuit (**e**), and its stretched part (**f**).

The PLA/rGO-SDBS composite was selected to print the models by FDM, due to its better electrical resistivity and thermal stability. As shown in Figure 13, the human-shaped phone stand holder and letters were printed in order to prove that complex shapes can be printed by the composite filament. The design of the phone stand holder before printing is shown in Figure 13a. In order to illustrate a clear view of the model, Figure 13b,c are presented, which are the full view and left view of the phone stand holder, respectively. The letters NEFU shown in Figure 13d represents the name of our university, Northeast Forestry University. Both the phone stand holder and the letters could be perfectly printed and the surface was smooth, proving that the composite is suitable for printing complex shapes. The circuit in Figure 13e was not broken during the stretching process (as shown in Figure 13f), suggesting that the composite has certain flexibility.

**Figure 14:**
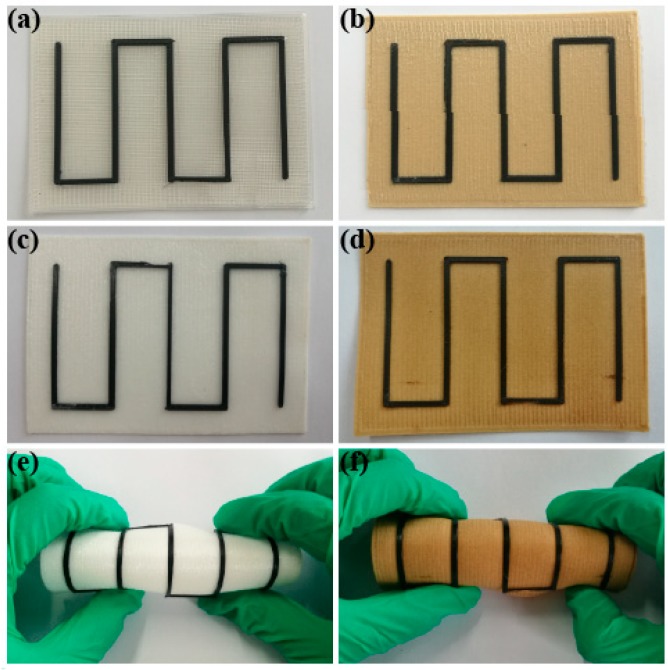
Patterns printed by FDM on different baseplates: patterns printed on PLA (**a**) and on PLA/wood flour composite (**b**); patterns printed on the flexible thermoplastic polyurethane (TPU) (**c**), on the TPU/wood flour composite (**d**), and their bent parts (**e**,**f**).

A two-layer pattern was printed on different baseplates, such as (a) on the PLA, (b) its composite with wood flour, (c) on the flexible TPU, and (d) its composite with wood flour. All materials used for the baseplates were prepared in our laboratory [9,47]. As can be seen in Figure 14, the patterns and the different baseplates could be satisfactorily combined and bent without separating from the baseplates. This also proved that the composite had certain flexibility.

## 4. Conclusions

The aim of this study was to improve the electrical conductivity of PLA-based composites. In order to improve the dispersion of the rGO or MWCNTs, a method of masterbatch melting was used to prepare composites, and TPU was used as a toughening agent for the PLA-based composites for the FDM technology. Four conclusions can be drawn from this study. Firstly, it was found that, by incorporation of 9 wt % rGO, the composites achieved a minimum volume electrical resistivity of 10^3^ Ω·m, which decreased by nine orders of magnitude compared with the control composite. Secondly, DSC results showed that the addition of rGO improved the cold crystallization ability of PLA, resulting in a decrease in the cold crystallization temperature. The composite also gained a better interfacial compatibility, apparent viscosity, and thermal stability. Thirdly, the functionalized rGO platelets were successfully prepared with TA, SDBS, and MWCNTs. The SDBS-functionalized rGO was found to be a more effective way of improving the electrical conductivity of the composite, since its resistivity dropped to 10^2^ Ω·m. Lastly, models and patterns printed by FDM demonstrated that the PLA/rGO-SDBS composite had a certain flexibility and was suitable for printing complex shapes. It could be printed into different baseplates fabricated in our laboratory, including virgin PLA and flexible TPU or their composites with wood flour.

## Figures and Tables

**Table 1:** The printing parameters for the fused deposition modeling (FDM) printer.

Nozzle Size	Layer Thickness	Nozzle Temperature	Print-Bed Temperature	Infill Density	Infill Pattern
0.8 mm	0.4 mm	210 °C	40 °C	100%	Linear

**Table 2:** Torque rheological parameters: the equilibrium torque (*T*_e_) and the shear heat (Δ*T*) of the redox graphene nanoplatelet (rGO)/polylactic acid (PLA) composites with different rGO contents.

Sample Codes	*T*_e_ (N·m)	Δ*T* (°C)
rGO-0	0.76	7.29
rGO-6	1.16	7.99
rGO-9	1.44	8.67
rGO-12	1.83	9.21

**Table 3:** Characteristic values of the differential scanning calorimetry (DSC) and thermogravimetric analysis (TGA) curves of the composites at different rGO loading.

Sample Codes	*T*_g_^1^ (°C)	*T*_m_^2^ (°C)	*T*_cc_^3^ (°C)	*X*_c_^4^ (%)	*T*_onset_^5^ (°C)	*T*_max_^6^ (°C)		Residue at 210 °C (wt %)	Residue at 600 °C (wt %)
						*T*_PLA_	*T*_TPU_		
rGO-0	60.35	167.11	103.35	49.80	278.49	301.97	344.63	99.69	2.08
rGO-6	60.05	167.21	102.22	44.48	274.73	299.67	365.95	99.39	7.46
rGO-9	60.04	166.79	100.62	45.81	281.25	302.52	363.00	99.20	8.44
rGO-12	59.72	166.82	100.75	47.77	289.52	310.06	360.53	99.49	10.17

^1^*T*_g_ represents the glass transition temperature of the composites; ^2^ *T*_m_ represents the fusion point of the composites; ^3^ *T*_cc_ represents the cold crystallization temperature; ^4^ *X*_c_ represents the crystallinity; ^5^ *T*_onset_ represents the epitaxial onset temperature; ^6^ *T*_max_ represents the temperature at which the maximum weight loss rate occurred.

## Appendix A

It can be observed from Figure A1 that, after sonication, a homogeneous black slurry can be obtained. After standing for 5 h at room temperature, the rGO in the unmodified slurry settled, but this did not occur in the modified slurry. This indicated that the stability of the slurry was improved after the functionalization of rGO.
